# The Role and Regulation of Thromboxane A_2_ Signaling in Cancer-Trojan Horses and Misdirection

**DOI:** 10.3390/molecules27196234

**Published:** 2022-09-22

**Authors:** Anthony W. Ashton, Yunjia Zhang, Rosanna Cazzolli, Kenneth V. Honn

**Affiliations:** 1 Division of Cardiovascular Medicine, Lankenau Institute for Medical Research, Rm 128, 100 E Lancaster Ave, Wynnewood, PA 19096, USA; 2 Division of Perinatal Research, Kolling Institute of Medical Research, Faculty of Medicine and Health, University of Sydney, Sydney, NSW 2006, Australia; 3 Departments of Oncology and Pathology (Bioactive Lipids Research Program), School of Medicine, Wayne State University, Detroit, MI 48202, USA

**Keywords:** thromboxane A_2_ synthase, thromboxane A_2_ receptor, isoforms, cancer, stroma

## Abstract

Over the last two decades, there has been an increasing awareness of the role of eicosanoids in the development and progression of several types of cancer, including breast, prostate, lung, and colorectal cancers. Several processes involved in cancer development, such as cell growth, migration, and angiogenesis, are regulated by the arachidonic acid derivative thromboxane A_2_ (TXA_2_). Higher levels of circulating TXA_2_ are observed in patients with multiple cancers, and this is accompanied by overexpression of TXA_2_ synthase (*TBXAS1*, TXA_2_S) and/or TXA_2_ receptors (*TBXA2R*, TP). Overexpression of TXA_2_S or TP in tumor cells is generally associated with poor prognosis, reduced survival, and metastatic disease. However, the role of TXA_2_ signaling in the stroma during oncogenesis has been underappreciated. TXA_2_ signaling regulates the tumor microenvironment by modulating angiogenic potential, tumor ECM stiffness, and host immune response. Moreover, the by-products of TXA_2_S are highly mutagenic and oncogenic, adding to the overall phenotype where TXA_2_ synthesis promotes tumor formation at various levels. The stability of synthetic enzymes and receptors in this pathway in most cancers (with few mutations reported) suggests that TXA_2_ signaling is a viable target for adjunct therapy in various tumors to reduce immune evasion, primary tumor growth, and metastasis.

## 1. Introduction

Eicosanoids are a group of oxygenated 20-carbon essential fatty acids, produced by phospholipid metabolism, that regulate various physiological pathways via autocrine and paracrine signaling [1,2]. Eicosanoids are primarily produced in response to stimulation and exhibit high potencies at low concentrations through the activation of cell surface or nuclear membrane receptors [1,2]. The primary source of pro-inflammatory eicosanoids is conversion of the omega-6 fatty acid (arachidonic acid) by the cyclo-oxygenase (COX) pathway. In contrast, most anti-inflammatory eicosanoids (including resolvins and protectins) are derived from omega-3 fatty acids, eicosapentaenoic acid (EPA) and docosahexaenoic acid (DHA) [2,3]. In the COX pathway arachidonic acid is converted to the intermediate metabolite prostaglandin G_2_ (PGG_2_), which reduces to PGH_2_. PGH_2_ is the central substrate for all prostaglandin synthesis with the formation of different molecular species through the action of terminal syntheses [2]. These prostanoids then mediate autocrine or paracrine responses by binding to their respective GPCRs [4]. Of the three COX isoforms COX-1 is constitutively expressed, whilst COX-2 is absent, in normal tissue [5]. Conversely, COX-2 is upregulated at sites of inflammation and by oncogenes, such as Ras and epidermal growth factor (EGF), in many cancers [4,5]. COX-3, a splice variant of COX-1, is most concentrated in the heart and cerebral cortex, and has a slower enzymatic activity than the other COX isoenzymes [5]. The first clues to the regulation of oncogenesis by eicosanoids came from the tumor suppressive effects of COX inhibitors (recently reviewed here [6,7]). COX-2 inhibitors (Celebrex and sulindac sulfide) inhibit the growth of prostate cancer xenografts and growth and metastasis of lung tumors in mouse models [8,9]. Moreover, long-term use of aspirin is associated with a 50% reduction in risk of endometrial cancer [10,11]. In preclinical models’ aspirin use markedly decreased the metastatic potential of colorectal cancer cells (HT29) [12]. These data suggest that a downstream metabolite of the COX pathway is vital for cancer progression.

## 2. Thromboxane A_2_ Biosynthesis

**Figure 1:**
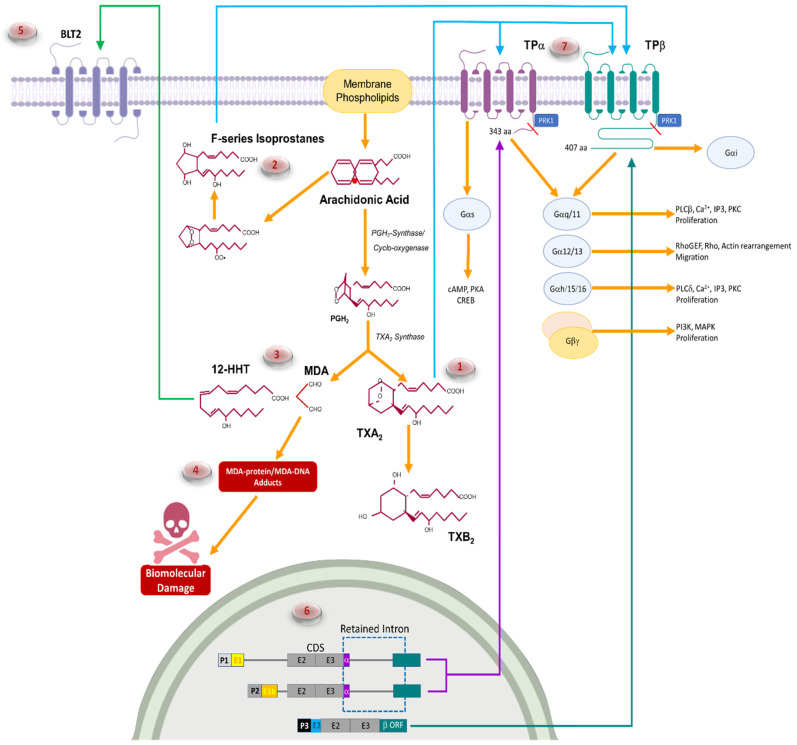
**Schematic for TXA_2_ biosynthesis and signaling.** Eicosanoid Synthesis (1) begins with the release of arachidonic acid from membrane phospholipids by phospholipase A_2_. Arachidonic acid is either metabolized to PGH_2_ by the cyclo-oxygenase enzymes or is attacked by oxygen free radicals to produce F-series isoprostanes (2). TXA_2_S then metabolizes PGH_2_ into either TXA_2_ (1) or 12-HHT and MDA (3). MDA forms DNA and Protein adducts to cause cellular damage and cell death (4) while BLT2 activation by 12-HHT enhances tumor formation (5). TP isoforms are derived from alternative splicing with TPα expression driven by promoter 1 and 2 (P1, P2) (6). TXA_2_ and F-series isoprostanes activate TP (7) to produce propagate carcinogenesis.

Thromboxane A_2_ (TXA_2_) was first identified in 1975 as being produced by platelets upon activation [13]. After liberation from membrane phospholipids by PLA_2_, arachidonic acid is metabolized by COX-1 and/or COX-2 enzymes to generate the prostaglandin endoperoxide, PGH_2_. PGH_2_ is metabolized to TXA_2_ by the terminal enzyme TXA_2_ synthase (TXA_2_S) (Figure 1(1)), an ER membrane protein that belongs to the P450 epoxygenase family [14]. TXA_2_ is unstable in aqueous solutions (half-life of 30 s) and spontaneously hydrolyses to the inactive TXB_2_ [13]. TXA_2_ acts primarily as a paracrine factor as activity requires constant synthesis. Indeed, TXB_2_ the degraded inactive product of TXA_2_, is often used as a marker when measuring TXA_2_ [13]. TXA_2_S was first found as a microsomal enzyme in platelets (60 kDa) but is highly expressed in lung, kidney, stomach, duodenum, colon and spleen [14]. TXA_2_ has biologically relevant roles in hemostasis (including platelet aggregation), vascular tone (contraction of vascular smooth muscle cells (VSMC), cell proliferation, and migration [15]. All these biological effects require TXA_2_ stimulation through a G-protein coupled receptor (thromboxane receptor (TP)), which is ubiquitously expressed on a variety of cell types [16].

However, additional potent bioactive by-products derived from TXA_2_ synthesis (Figure 1(3)), malondialdehyde and 12-HHT are made in equimolar amounts along with TXA_2_ [17]. The current model for how this occurs suggests interaction of the heme group of TXA_2_S with the C-9 oxygen of PGH_2_. Homolytic scission of the endoperoxide bond, immediate formation of the alkoxy radical and “β-scission” of the C11-C12 bond yields an allylic radical which, alternatively, decomposes into HHT and malondialdehyde, or undergoes Fe(IV)-oxidation and ionic rearrangement to TXA_2_ [18]. However, TXA_2_S metabolizes other endoperoxide substrates (PGH_1_, 8-iso-PGH_2_, or 15-keto-PGH_2_) producing MDA and the corresponding C17 metabolite (i.e., HHD, 8-cis-HHT and KHT) without substrate preference [17]. Free radical fragmentation into HHT and MDA requires only the Fe(II) or Fe(III) for catalysis. Thus, the heme ferric iron of thromboxane synthase (and other cytochrome P-450 members) is sufficient [17]. Metabolism of these alternate substrates does not result in TXA_2_ synthesis [17,18] but suggests catalysis of MDA and bioactive C17 lipid moieties by TXA_2_S is a more significant modulator of cell behavior than expected. 12-HHT exerts its biological effects through the BLT2 receptor [19], which potently affects carcinogenesis (see below).

Yet, another TP ligand is produced from arachidonic acid, F-series isoprostanes (Figure 1(2)). Isoprostanes are produced from unsaturated fatty acids in a predominantly COX-independent manner [20]. In contrast to COX generated prostaglandins derived from free arachidonic acid, isoprostanes are initially formed from arachidonic acid esterified in membrane phospholipids. F_2_-isoprostanes are released from the phospholipid backbone as free fatty acids by phospholipases. Isoprostanes are formed when an oxygen free radical mediates a nucleophilic attack on a carbon flanked by double bonds [20]. The free radical intermediate reacts with oxygen to form a peroxyl radical (LOO-), which reacts on either face of the sidechain with a further oxygen molecule to produce a racemic hydroperoxy bicyclic endoperoxy radical [20]. Finally, the radical chain reaction is terminated by abstraction of hydrogen from an appropriate donor molecule (such as another polyunsaturated fatty acid). The product is an isoprostane-prostaglandin, an analogue of PGG_2_, which is reduced to the stable F_2_-isoprostanes. The central structural distinction between isoprostanes and cyclooxygenase-derived prostaglandins is that the former contain sidechains that are predominantly oriented cis to the prostane ring, while the latter possess exclusively trans sidechains [20]. The formation of F-series isoprostanes are independent of enzymatic properties and reliant only on s source of free radicals and phospholipids. As such, isoprostanes are frequently found in hypoxic environments and conditions promoting mitochondrial dysfunction. As such, isoprostane and TXA_2_ generation can occur completely independently in the same tissues without interference. F_2_-isoprostanes are thought to exert their biological activity, at least in part, through TP isoforms, as TP antagonists block the platelet activation and vasoconstriction of vascular smooth muscle cells/carotid arteries in response to 8-iso-PGF_2α_ [21].

## 3. Thromboxane A_2_ Receptor Isoforms and Signaling

The activation of cells by TXA_2_ is mediated by T-prostanoid receptors (TP; Figure 1(7)), which are G-protein coupled receptors on the cytoplasmic membranes of all responsive cells. In humans there are two TP isoforms, TPα [22] and TPβ [23,24], coded by alternative splicing of a single gene on chromosome 19.13.3 [25] (Figure 1(6)), Table 1). The two isoforms are identical in their N-terminal 328 amino acids. At this point, the two isoforms diverge to produce proteins with cytoplasmic tails of 15 and 79 amino acids for TPα and TPβ, respectively [23,24] (Figure 1(7)). The TP gene (*TBXA2R*) contains 4 exons and 3 introns with alternative splicing in exon 3 producing TPα and TPβ [26,27] (Figure 1(6)). A significant determinant of splicing is the promoter region used to generate the mRNA transcript. Three promoters regulate TP gene transcription, with transcripts derived from promoter 1 and 2 remaining unspliced to produce TPα [26,27]. Conversely, promoter 3 derived transcripts are spliced to produce TPβ [26,27]. The transcripts are otherwise identical suggesting that the 5’untranslated region from promoter 3 may contain nucleolar targeting sequences or sequences that attract components of the splicosome apparatus that remove the retained intron and promote TPβ expression (Figure 1(6)). TP in most reported species thus far is an orthologue of TPα. While TP expression has been identified in a wide range of cell types and organs, the distribution of the two TP isoforms has not been as well documented, most likely due to complacency over the similar function of the two receptor isoforms.

TPα is the dominant TP isoform [16] in all tissues examined thus far, including vascular and uterine smooth muscle, endothelial cells, trophoblasts, platelets, brain, thymus, intestine and liver [16] and cancer cells from many organs (see below). Conversely, TPβ expression is documented in a few of these tissues to a significant degree. Our studies have shown robust TPβ expression in endothelial cells, which profoundly affects angiogenic potential (see below). Variations in TPβ expression between fetal and adult tissues may exist with TPβ expression lost after birth [16]. In addition, the reported expression of TPβ in platelets by mRNA was not replicated with IHC/immunoblotting [28], suggesting that low levels of mRNA transcripts may not be adequate for robust protein expression in all tissues.

The divergent cytoplasmic tails of the TP isoforms are not significant discriminators of ligand binding, as TXA_2_ has similar affinity for both isoforms. However, maximal biological activity of F2-series isoprostanes requires TPβ expression, suggesting some selectivity [21] (Figure 1(7)). The relative ligand preference of TP is not determined; however, TP can be re-challenged by U46619 after isoprostane stimulation, but the reverse is not true. [29] In addition, TPα (PTB3 [30],16f [31]) and TPβ (BM573 [29], 9h, 9af, and 9ag [31,32])-specific antagonists have been rationally designed, indicating that the intramolecular interactions of the TPβ cytoplasmic tail produce structural rearrangements of the ligand binding site that allow for ligand discrimination. The C-terminal cytoplasmic domains also do little to discriminate G-protein coupling of the two isoforms (Figure 1(7), Table 1), with both isoforms coupled to Gαq, Gα11, Gα12, Gα13, Gα15, and Gα16 [15,33]. Vezza and colleagues [34] found that only TPα signals through Gαh, which is associated with cellular survival mechanisms and the activation of phospholipase C (PLC)-dependent inositol phosphate (IP) formation. Similarly, TPα activates adenylate cyclase to synthesize cyclic adenosine monophosphate (cAMP), while TPβ inhibits adenylate cyclase activity [35]. This suggests that TPα couples with Gαs and TPβ with Gαi [24,35]. Both isoforms depend on Src-kinase to phosphorylate ERK, while the transactivation of EGFR through TP activates the PI3K-Akt phospholipase Cγ1 (PLC-γ1) pathways, which increase survival and migratory potential, respectively [36].

**Table 1:** Divergent biological properties of the two human TP isoforms.

Receptor Property	TPα	TPβ
Length of cytoplasmic tail	15 amino acids [22]	79 amino acids [37]
Specific 2nd messengers	Gαh [34], Gαs, stimulates cAMP [35].	Gαi, Inhibits cAMP [35]
Response to Stimulation		
Acute	Phosphorylated, not internalized [38,39]	Internalized in a GRK, and arrestin dependent manner [39]
Chronic	Decreased expression [38]	Enhanced expression [38]
Desensitization		
Phosphorylation Sites	Ser^329^ [40,41] and Ser^331^ [42,43]	Thr^399^ [44]
Kinases or agonists involved	Ser^329^: PGD_2_ [40], PGI_2_ [41,42], PGE_2_ [43], and cAMP/PKA [45] Ser^331^: NO/PKG [42,43]	PKC-α [38] and G-protein receptor kinases 2, 5 and 6 [39]
Resensitization to ligand	Dephosphorylation phosphatases PP1 and PP2A [46]	Recycling of the receptor to the surface after lysosomal-mediated ligand degradation [39]

Conversely, the divergent residues of the two TP isoforms produce very different post-translational modifications, protein binding and inactivation mechanisms after ligand binding. TPβ, but not TPα, undergoes agonist- and tonic-induced cellular internalization that recycles the receptor to the cell surface and re-sensitizes the response to the ligand [39,47]. Agonist-induced desensitization of TPα is achieved through phosphorylation of residues Ser^329^ [40,41,45] and Ser^331^ [42,43] whilst re-sensitization involves de-phosphorylation of these same residues by PP2A [46]. Conversely, agonist-induced desensitization of TPβ involves phosphorylation by GRK 2, 5 and 6 and subsequent internalization through interaction with dynamin and arrestin [39], Rab11 [48] and Nm23-H2 [49]. In addition, palmitoylation of TPβ at Cys^347^ is required for Gq coupling and activation of PLCβ whilst palmitoylation of TPβ at Cys^373^/Cys^377^ is needed for TPβ internalisation [47]

## 4. TXA_2_ Signaling in Cancer

The prominence of TXA_2_ signaling in processes such as atherosclerosis, infarction, hypertension, stroke and renal dysfunction has resulted in effective antagonists central to the therapy of these diseases. Recently, multiple studies have indicated functional roles for both *TBXAS1* and TP in the essential processes of neoplastic transformation including enhanced tumor cell motility and invasion, proliferation, and therapeutic resistance that are critical steps in cancer progression [50]. These effects are observed in multiple cancers indicating the profound effects on tumorigenesis and the widespread clinical applicability of targeting TXA_2_ signaling as adjunct therapy for cancer.

### 4.1. TP Isoforms in Cancer

**Figure 2:**
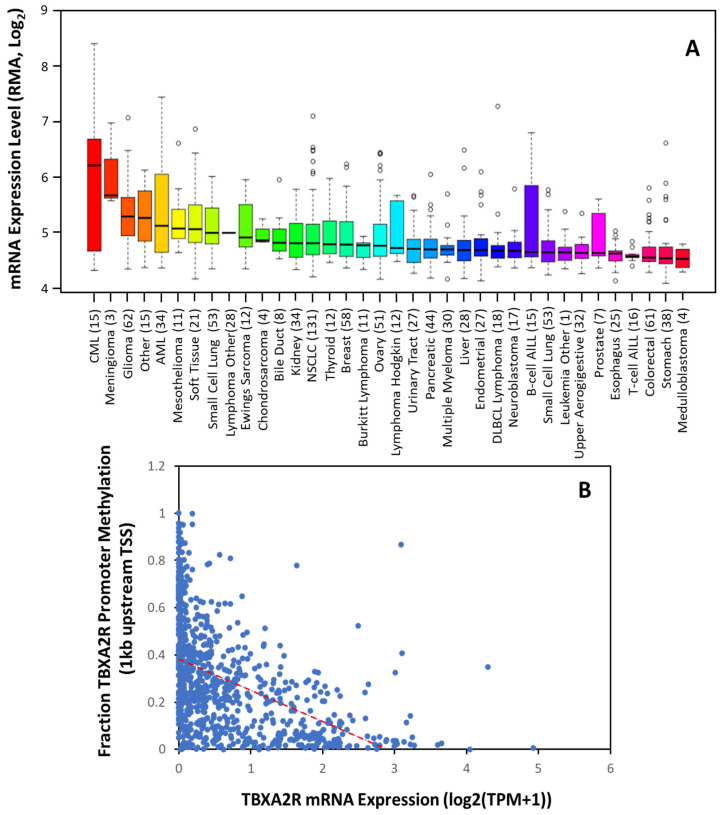
***TBXA2R* Expression is Increased in The Majority of Tumors.** (**A**) Expression of *TBXA2R* mRNA in 36 different cancer cell types. Data are mean ± SD showing 95% CI. Numbers above descriptors on the *X*-axis denote the number of individual cell lines from which the data were derived. (**B**) TCGA data from 59,132 patients’ samples showing the correlation between *TBXA2R* mRNA expression and methylation of the promoter (within 1 kb of transcriptional start site (TSS)). Red dotted line denotes linear correlation (R^2^ = 0.442).

Thromboxane receptors are upregulated in multiple tumors, including multiple myeloma, skin, prostate, breast, lung, colon, bladder, and brain cancer [50]. Consistent with this, The Cancer Cell Line Encyclopedia shows significant upregulation in these cancers (Figure 2A). Moreover, the data suggest *TBXA2R* expression is elevated to a greater extent in some cancers (such as CML, meningioma, AML, mesothelioma and renal cancer) where the significance is yet to be determined. Transcription control is the primary mechanism of regulation over *TBXA2R* expression in most cancers. Sp1 activates the transcription of TPα at promoter 1, whilst Egr1 impedes its transcription by competing with Sp1 at overlapping Sp1/Egr1 sites in HEL cells [51]. NF-E2, GATA-1 and Ets-1 were also implicated in the transcriptional regulation of TPα in megakaryocyte differentiation and human platelets [51]. A recent study found that a BRCA1-c-Myc complex transcriptionally represses TP gene expression and that a BRCA1 knockdown in an ER^+^PR^+^ cell line, T-47D, upregulates *TBXA2R* expression [52]. Other tumor suppressor genes, Wilms’ tumor (WT1) and hypermethylated in cancer (HIC1) also repress TPα expression by binding to promoter 1 in breast and prostate cancer cell lines [53] to regulate basal expression of TPα.

Using the non-redundant pan-cancer studies database (TCGA, 32 cancer types, 59, 132 patients), we determined that TP mRNA expression is inversely correlated with *TBXA2R* promoter methylation across 23 different cancers. Unfortunately, showing decreases in a global analysis (Figure 1B) is difficult, as the ubiquitous expression of TPα results in low promoter methylation in most tissues. However, these data agree with previous findings that promoter 1 is hypomethylated in benign and precursor lesions, but undergoes increasing methylation with prostate cancer staging [54]. Thus, TP isoforms may be transcriptionally regulated; however, epigenetic modifications during carcinogenesis (such as promoter methylation) play an equally important role in determining expression [54,55].

The main cellular activities induced by TP over-expression to promote cancer are increased proliferation, migration, and invasion. Moreover, antagonizing TP re-sensitizes cancer cells to more conventional chemotherapy [56] and promotes responsiveness to drugs where resistance has developed. TPα and TPβ both activate extracellular signal regulated protein kinase (ERK) and phosphatidylinositol 3’kinase (PI3’K) signaling. TP activation in human astrocytoma [57], bladder [56] and prostate cancer [58,59] cells induces morphological change, enhanced motility, invasion and metastasis via the Gα12/RhoA pathway. TP activation induces DNA synthesis by activating ERK via Gαq/11 signaling [57]. In bladder and prostate cancers, TP-mediated ERK activation phosphorylates the tumor suppressor protein forkhead box O3 (FOXO3), which is deacetylated by SIRT1, resulting in Skp2-mediated degradation. The loss of FOXO3 was linked to the enhanced migration and invasion in both cancer cells [60,61,62]. Coupling to Gαh/15/16 is associated with cellular survival mechanisms, and activation of phospholipase C (PLC)-dependent inositol phosphate (IP) formation and PI3K activity, both of which influence tumor cell proliferation/mitogenesis [15]. Coupling of TPα to Gαs in 4-methylnitrosamino-1–3-pyridyl-1-butanone induced lung cancer stimulates PKA/CREB activation [63], resulting in expression/activation of the orphan nuclear receptor Nurr1, which stimulated proliferation of human lung cancer cells but could also be implicated in differentiation and apoptosis [63,64]. Moreover, the interaction of the receptors with non-traditional downstream regulators has significant consequences for cancer progression. The interaction of both isoforms with the protein kinase C-related kinase (PRK)-1 and PRK-2 [65,66] was essential for TP-induced prostate cancer cell migration, but also enabled TP activation to manipulate histone H3 phosphorylation at Thr11 (H3Thr^11^), an epigenetic marker both necessary for and previously exclusively associated with androgen-induced chromatin remodeling and transcriptional activation. The significance of TP-PRK interactions have not been described outside of prostate cancer; however, TP-PRK1/2 signaling in other cancers could explain the aggressive phenotypes associated with high TP expression [50].

**Figure 3:**
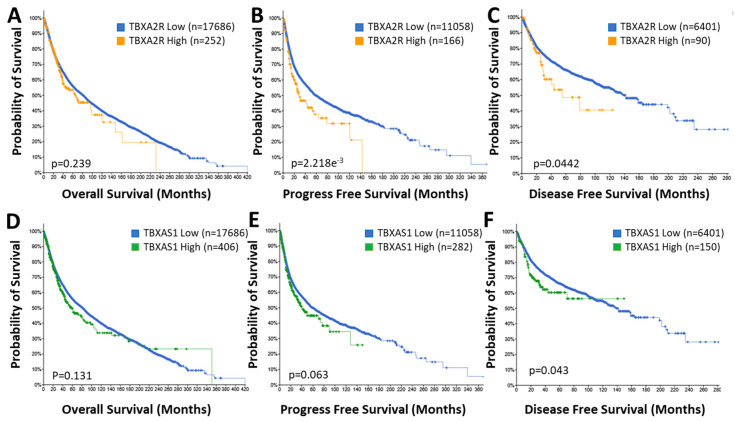
**Elevated *TBXA2R* and *TBXAS1* Expression Is Associated with Poor Prognosis.** Data from the TCGA pan-cancer database for *TBXA2R* (**A**–**C**) and *TBXAS1* (**D**–**F**) was correlated with overall (**A**,**D**), progression free (**B**,**E**) and disease free (**C**,**F**) survival in patient populations with low (♦) and high (♦, *TBXA2R*; ♦, *TBXAS1*) expression. n = number of patients in the cohort.

TP expression is correlated significantly with poorer outcomes in lung [67], breast [68,69], bladder [56], colorectal [70] and prostate [71] cancers. TP expression is often higher in advanced tumor stages (such as higher Gleason score in prostate cancer) and metastatic disease and is associated with important clinical endpoints, such as reduced disease-free survival [68,69]. Using the non-redundant pan-cancer studies database, we established that patients with high *TBXA2R* expressing tumors have substantially poorer outcomes concerning progress free (29.49 vs. 58.72 months; *p* = 2.218 × 10^−3^) and disease free (55.04 vs. 140.90 months; *p* = 0.0442) survival (Figure 3B,C) but not overall survival (Figure 3A) compared to patients with low expression. Significantly, 5-year progression free survival for the *TBXA2R* high group was lower (38%) compared to the *TBXA2R* low group (49%) and total lifespan was no more than 140 months (360 months for the *TBXA2R* low group) (Figure 3B). Similarly, 5-year disease free survival for the *TBXA2R* high group was 48% (compared to 67% for the *TBXA2R* low group) with overall longevity reduced from 280 to 120 months (Figure 3C). Even overall survival (although not significantly different between the two groups) was reduced (420 months down to 230 months) in the *TBXA2R* high group (Figure 3A). These data indicates the profound impact of TP expression on cancer progression and highlights the value TP antagonists might bring to cancer therapy.

The significance of individual TP isoforms in cancer has been poorly investigated. TPα is expressed by most non-transformed epithelial cells and is almost exclusively the only TP isoform expressed in most solid tumors (including lung, NSCLC and small cell, prostate cancer) [50], while TPβ drives the progression of bladder cancer [56]. In bladder cancer, increased expression of TPβ, but not TPα, was observed in epithelial and stromal cancer tissue [56]. TPβ overexpression was correlated to increased proliferation and increased metastatic potential through Gα12/13 signaling and induced malignant transformation in xenografts of normal bladder epithelial cells *in vivo* [56]. Moreover, elevated TPβ expression was associated with shorter disease-free survival time in patients [56].

Bladder cancer is rare, as roles for both TP isoforms have been identified and provides evidence for the distinct roles for TPα and TPβ in cancer pathogenesis. TPβ is derived from the activation of promoter 3, which, unlike promoter 1, is hypermethylated in benign and precursor lesions and becomes increasingly hypomethylated with the increase in tumor grade, leading to increased TPβ expression [54]. TPβ expression is negatively regulated through promoter 3 by peroxisome proliferator-activated receptor (PPAR)γ activation with 15-deoxy-D12,14-prostaglandin J2 [72,73]. Furthermore, promoter 3 is activated through activator protein-1 (AP-1) and OCT-1/-2 binding elements [26]. In addition, oxidative stress promotes the translocation of TPβ from the endoplasmic reticulum to the Golgi complex and ultimately into the plasma membrane [74,75]. Oxidative stress induces maturation and stabilization of the TPβ protein, prolonging protein half-life. Given the elevated oxidative stress observed in some tumors [76], protein stabilization likely plays a role in tumors where TPβ expression is enhanced. Finally, a recent phenome-wide association study examined the association of the rs200445019 polymorphism in *TBXA2R* (T^399^A substitution in TPβ that impairs ligand induced desensitization) with phenomic outcomes [77]. Surprisingly this study significantly associated The TPβ (T339A) mutation with metastatic disease at multiple tissue sites (including lymph nodes, respiratory organs, digestive systems, brain/spine) derived from various solid tumor types, including breast, colon, lung, head and neck, renal, gastric and ovarian cancers [77]. Based on this analysis Kaplan–Meier analysis associated high *TBXA2R* with poor prognosis and reductions in median disease-free survival in patients with breast, head and neck, lung, ovarian, esophageal, renal, pancreatic and gastric cancers (all of which would have been predicted by Figure 2A) [77]. What is exciting about these data is that the complement of TP isoforms expressed in many of these cancers have not been documented; however, based on the phenomic analysis, it is likely that TPβ expression might be upregulated in at least breast, colon, lung, head and neck, renal, gastric and ovarian cancers [77]. This result indicates isoform switching could be a widely used molecular event in cancer pathogenesis and may represent a key targetable pathway during tumorigenesis. The data seem to highlight that TPα and TPβ may each play a role in tumor growth/development, depending on the cell and tissue involved.

### 4.2. TBXAS1 in Cancer

Increased circulating TXB_2_ is found in the serum of patients with lung cancer and peritumoral tissue surrounding laryngeal cancers, compared to healthy mucosa. Tissue samples of non-small cell lung carcinoma had higher levels of TXB_2_, compared to non-cancerous tissue, which positively correlated with the disease stage (i.e., more advanced cancer samples had higher levels of TXB_2_). High levels of TXB_2_ in lung cancer tissue was associated with a high level of lipid peroxidation and Bcl-2 expression [78]. However, a direct link between these signaling molecules is yet to be established. Karmali et al. found high TXB_2_ levels associated with large tumors and lymph-node metastases in breast cancer [79] In addition, elevated urinary protein levels of TXB_2_ may prove a valuable prognostic and diagnostic tool in bladder cancer [80].

**Figure 4:**
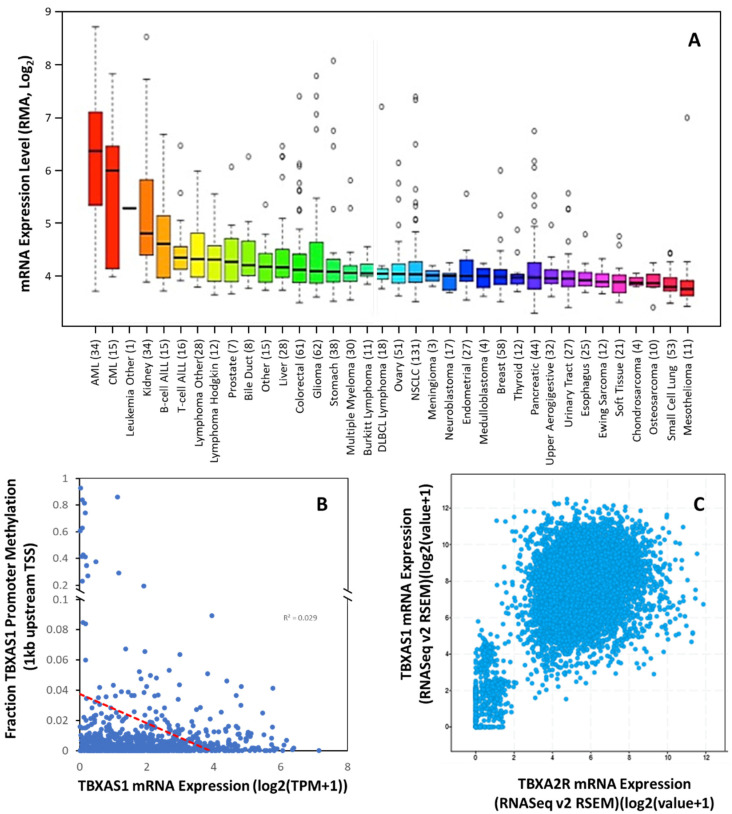
***TBXAS1* Expression is Dysregulated in Multiple Tumors.** (**A**) Expression of *TBXAS1* mRNA in 36 different cancer cell types. Data are mean ±SD showing 95% CI. Numbers above descriptors on the *X*-axis denote the number of individual cell lines from which the data were derived. (**B**) TCGA data from 59,132 patient samples showing the correlation between *TBXAS1* mRNA expression and methylation of the promoter (within 1 kb of transcriptional start site (TSS)). Red dotted line denotes linear correlation (R^2^ = 0.029). (**C**) Correlation of *TBXA2R* and *TBXAS1* expression for cancers in the TCGA pan cancer dataset.

Overexpression of TXA_2_S and increased levels of TXA_2_ have been demonstrated in thyroid [81], colorectal [82], bladder [83], lung [63,67,84], prostate cancer [71], and NSCLC [85] and renal cancer (Figure 4A). Cells overexpressing TXA_2_S grow at an accelerated rate and exhibit greater resistance to apoptosis [67,85,86,87,88,89,90], and TXA_2_S was needed for the development of metastasis [91,92,93,94], suggesting that enhanced TXA_2_ levels due to TXA_2_S overexpression activate the TP-dependent pathways of carcinogenesis. High levels of TXA_2_S expression were reported in glioma cell lines and in biopsies from glial tumors when compared to normal brain tissue, with expression levels positively correlated to cellular migration rates [95]. Schauf and colleagues determined that TXA_2_S, activity was essential to radiation insensitivity in glioma cells, which was subsequently proven in an orthotopic glioblastoma mouse model where TXA_2_S antagonism with furegrelate significantly reduce tumor size, slowed tumor cell proliferation, decreasing angiogenesis and increased apoptotic cell death [96]. In prostate cancer, *TBXAS1* expression is correlated with the severity of prostate carcinoma lesions, with advanced stages and poorly differentiated forms having the highest expression levels [71]. The enzyme was involved in motility, but not proliferation or survival, of prostate cancer cells [71]. In breast cancer, there is dissent over the correlation of *TBXAS1* expression and tumor grade, with one study [68] suggesting loss of expression with increasing grade but the reverse has also been reported [69]. However, *TBXAS1* polymorphisms have shown a modest association with breast cancer risk and poor outcomes [97].

Like TP expression, The Cancer Cell Line Encyclopedia suggests the addition of cancers with significant *TBXAS1* expression that are currently uncharacterized, including ALL, AML, CML, and renal cancer. Of these cancers, at least AML and CML also had significant *TBXA2R* expression (Figure 2A). Indeed, this phenomenon has been previously reported in a few cancers (bladder, breast, prostate) but has not been universal. What was not previously apparent is the high degree of correlation between *TBXA2R* and *TBXAS1* expression across all tumors in the TCGA pan-cancer dataset (Pearson co-efficient 0.58; *p* < 0.0001)(Figure 4C). These data suggest co-regulation of *TBXA2R* and *TBXAS1* is common in multiple types of cancer.

Like TP expression, increased TXA_2_S expression in cancer is transcriptionally driven. The *TBXAS1* promoter has two motifs in common with other cytochrome p450 enzymes: TATA independent transcription and multiple transcriptional start sites [95]. Maximal promoter activity resides within the first 285 bp. Two clusters of positive regulatory elements (PRE1 (−90 to −50 bp) and PRE2 (−50 to −25 bp)), accounting for ~75% of promoter activity, are counter-balanced by repressive elements between −365 and −665 bp [98]. While similar nuclear factor(s) from different cell types interact with PRE2, those interacting with PRE1 exhibit cell specificity. The three original cell lines containing PRE1 binding proteins were all leukemic, whilst cells not utilizing PRE1 were “normal” (CHO and murine macrophages) [98]. Utilization of PRE1 by cancer cells might explain the induction of TXA_2_S in multiple tumor types. The other essential regulatory site was between (−60 to −50 bp), the deletion of which compromised *TBXAS1* promoter activity [98]. Originally thought to be an AP-1 site, this site turned out to be trans-activated by NF-E2. Interestingly, many of the cancers with most significant TXA_2_S expression also highly express NF-E2, including bladder, invasive breast, lung, colon and prostate cancers (TCGA data). However, in NSCLC and small-cell lung cancer cells, TXA_2_S expression is regulated by NF-κB [67], suggesting unrecognized transcription factors may play a role in regulating *TBXAS1* transcription in cancer.

There is reported epigenetic regulation of *TBXAS1* expression. Our analysis of human methylation in the *TBXA2R* promoter (first 1 kb) revealed little evidence of epigenetic regulation in cancer, as the correlation of mRNA and promoter methylation is relatively poor (R^2^= 0.029) (Figure 4B). Other evidence from the 5.5 kb promoter suggests that distant repressive elements of the promoter (−3400, −3000, and −1430 bp) are methylated (including the long inter interspersed element). No promoter methylation was observed in cells without TXA_2_S expression; however, complete methylation at −1430 (and partial methylation at sites −3400 and −3000) was associated with TXA_2_S expression [98]. Whether these cell-type specific epigenetic mechanisms play a role in the induction of TXA_2_S in cancer have yet to be determined but given the perturbations in epigenetic regulation that exist in cancer, and the previous use of such mechanisms in leukemic cells, it is a high probability that they do.

While the trend for increased TXA_2_S expression in multiple cancers is well-established, the prognostic value of the findings are less clear. In bladder and prostate cancer patients, overexpression of TXA_2_S was associated with reduced overall survival [71,83]. However, data in breast cancer patients are less clear, with TXA_2_S expressed at significantly lower levels in patients with high grade tumors with poor prognostic outcome [68] in one study but high *TBXAS1* expression correlated with invasive disease and higher tumor grades in another [69]. Furthermore, Cathcart and colleagues observed no prognostic role for *TBXAS1* in NSCLC, despite significant elevations in *TBXAS1* (and TXB_2_) levels in tumor tissues than the matched “normal” tissues [85]. This confusion becomes more apparent when assessing the TCGA pan-cancer dataset for correlations between *TBXAS1* expression and clinical outcomes (Figure 3D–F). Like the data for *TBXA2R*, the overall survival of patients with high expression of *TBXAS1* in their tumors is not significantly affected, nor is the progression free survival (although at *p* = 0.063 it is close to a significant correlation). However, there is a substantial reduction in disease free survival in the *TBXAS1* high group, although the effect size is not as large as that for *TBXA2R*. Five-year disease-free survival is reduced from 67% in the low group to 60% in the *TBXAS1* high group. However, overall longevity of the disease-free cohort is still decreased (from 280 to 150 months) by high *TBXAS1* expression. While the jury might still be deliberating the utility of TXA_2_S expression as a prognostic marker, there is no questioning it is elevated in cancer and antagonists of TXA_2_S potently manipulate cancer progression.

## 5. Trojan Horses: The By-Products of Txa_2_ Biosynthesis in Cancer

As stated earlier, TXA_2_ biosynthesis also generates malondialdehyde and 12-HHT in a 1:1:1 molar ratio [17]. Whilst the focus of TXA_2_S inhibitors has focused on the lack of TXA_2_ synthesis, to date, no studies have assessed the reduction in either malondialdehyde or 12-HHT as part of the resulting anti-tumor phenotype. Malondialdehyde (MDA) can react with DNA and protein to form stable adducts. Under physiological conditions MDA forms adducts with deoxyguanosine and deoxyadenosine, with the principle product being 3-(2-deoxy-β-d-erythro-pentafuranosyl)pyrimido [1,2-α]purin-10(3H)-one deoxyguanosine (M1dG) [99]. If not repaired, M1dG is a mutagenic lesion that produces both base pair substitutions (G → T and G → A) and frameshift mutations in DNA associated with carcinogenesis and contributes to the etiology of human cancer. Moreover, MDA reacts with the ε-amino groups of lysine residues in proteins, resulting in adducts with several structures, including the formation of intra- and inter-molecular cross-links and covalent post-translational modifications that manipulate protein function [100,101]. Indeed, Dilysyl-MDA crosslinks are formed in activated platelets in a COX-TXA_2_S manner and are enhanced in diseases associated with enhanced platelet aggregation [102]. Such MDA-protein-like adducts modify proteins to change their activity, with significant impacts on chemo-resistance (AKR1B10, GST), cell growth (Pin1, EGFR, PPAR), survival (GCL, proteasome, PPAR) and metastatic capacity(α-elonase) in cancers such as glioma, leukemia, breast, renal and colon cancer. Moreover, MDA-protein adducts modulate the tumor microenvironment to promote cancer progression (as reviewed in [103]).

The metabolite 12-HHT also plays a role in carcinogenesis by activating its cognate receptor BLT2 [19]. Levels of 12-HHT in activated platelets from *TBXAS1* null mice are reduced by greater than 80% compared to wild-type mice, suggesting that most HHT produced in cells is TXA_2_S-derived [104]. Under physiological conditions, BLT2 activation maintains epithelial barrier function in organs such as skin, colon and kidney, with BLT2 overexpression increasing barrier function and BLT2-null mice prone to trans-epidermal water loss and delayed cutaneous wound healing [105]. Enhanced BLT2 expression is observed in TNBC, thyroid follicular, renal, bladder, esophageal, colon and ovarian serous carcinoma versus normal epithelium [106]. BLT2 activation is associated with enhanced proliferation, survival and invasion in TNBC and bladder cancer cells [107,108], is required for Ras-induced transformation [109] and increased distant metastasis in a patient xenograft and orthotopic metastasis models in mice [110]. Indeed, breast cancer patients with high BLT2 expression had a lower disease-free-survival rate [107]. Pharmacological inhibition of BLT2 in these models prevented metastasis, and siRNA targeting of BLT2 prevented invasion of breast cancer cells *in vitro* [110,111]. Thus, in addition to TXA_2_, both MDA and 12-HHT contribute to tumorigenesis and collectively suggest TXA_2_S as a prime target for anti-cancer therapies.

## 6. TXA_2_/TP Antagonists as Adjuvant Therapies

**Figure 5:**
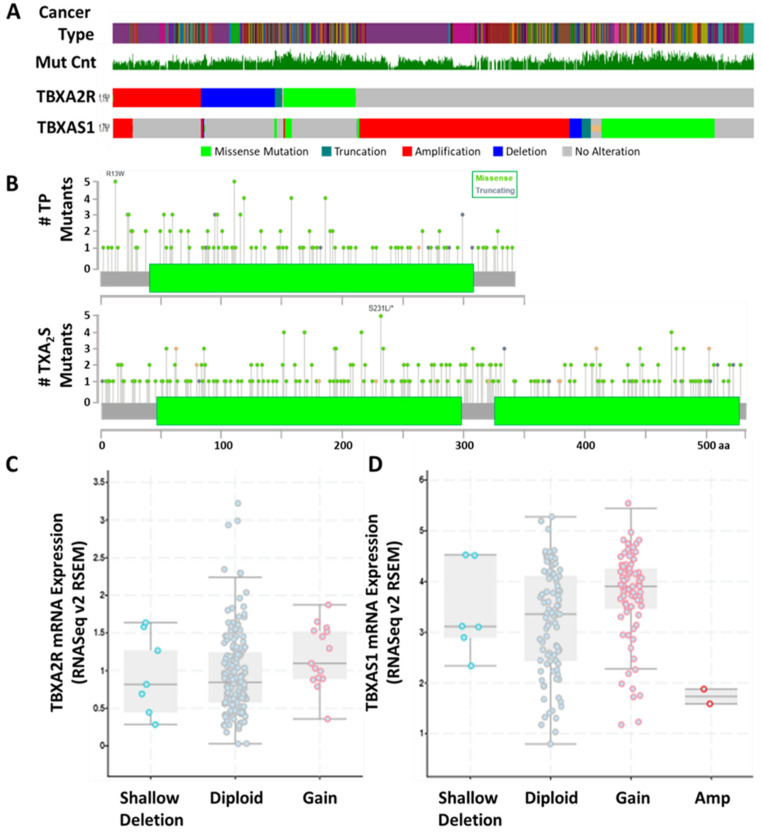
***TBXA2R* and *TBXAS1* are Genetically Stable Targets for Anti-Cancer Therapy.** (**A**) Mutational analysis of *TBXA2R* and *TBXAS1* mutants from the TCGA pan-cancer dataset. The different types of cancer in the analysis and the average total mutation count (MutCnt) for each patient are shown. Gain (■) and loss (■) of copy number are differentiated from missense (■) and frameshift (truncation, ■) mutations. (**B**) Schematic of TPα and TXA_2_S showing the position of the most frequent mutations and how many times they occurred in (A). The 7 transmembrane regions of TP and active domains of TXA_2_S (■) are shown for reference. (**C**,**D**) Correlation of change in copy number with mRNA expression for *TBXA2R* (**C**) and *TBXAS1* (**D**) indicates whether changes in copy number are required for changes in transcription of both genes in cancer.

Cancer is a problematic condition from a therapeutic viewpoint. Therapy resistance to treatments such as chemotherapy, radiotherapy and targeted therapies is a significant problem, with the mechanisms producing insensitivity resulting in more aggressive clones that contribute to poor prognosis [112]. In part, resistance mechanisms are driven by genomic instability, high mutation rates, and epigenetic changes in the cancer cell and/or the tumor microenvironment, with differences observed between early and advanced stages of cancer [112]. Mutation rates of TP and TXA_2_S in the TCGA pan-cancer dataset suggests that both targets are poorly mutated in cancer (1.1% for *TBXA2R* and 1.7% for TXAS1) (Figure 5A). When examining the specifics of the mutations, the changes in the TXA2R gene are almost evenly split between (36%, ■), deletion (30.6%, ■) and missense/truncating mutations (29.9%, ■/■). Truncation mutations in TP producing tail-less truncation mutants would still be functional and contribute to carcinogenesis. Conversely, all other truncation mutations would produce non-functional (potentially unstable) receptors, as would the point mutations at R60 and E129. All other mutations are of unknown consequence but are not predicted to be driver mutations. Mutation of *TBXAS1* in this pan-cancer panel is, philosophically, quite different (Figure 5A). For *TBXAS1*, 61.7% of all mutations are amplifications, which may contribute directly to the overexpression of this gene in many tumors, while only 3.5% of changes are deletions and 32.3% are missense/truncating mutations. Similar to *TBXA2R* mutations, most missense mutations in *TBXAS1* are of unknown consequence and not predicted to be driver mutations. Given that these genetic alterations are in a minority of tumors, it is perhaps not surprising that the correlation between mRNA expression and allele number suggests that the enhanced expression of TP and TXA_2_S is derived primarily from diploid tumor cells (Figure 5C,D). These data indicate that TXA_2_ signaling provides a stable target in a landscape of perpetual phenotypic and genomic instability.

The susceptibility of tumors to approaches that diminish TXA_2_ generation and signaling has been documented *in vitro* and in preclinical models where they yield significant therapeutic advantage. TXA_2_ antagonism with shRNA/CRISPR knockdown approaches [63,77,83,90,95,113,114] in multiple tumor types (colorectal, bladder, brain, lung cancer, prostate, myeloma) decreases proliferation, motility, invasion, anchorage-independent growth, and promotes apoptosis (either as solo therapy or to sensitize cells to chemotherapeutics (such as carboplatin and cisplatin)). Inhibition of TXA_2_S with selective inhibitors induced apoptosis (via overproduction of ROS and reduction of NF-κB activity), confirming that it is indeed a potential therapy target in NSCLC [85,113]. Conversely, the overexpression of TP or TXA_2_S [69] or use of TP agonists [63,83] has the opposite effects and exacerbates the transformed phenotype.

*In vivo*, TP antagonists/dual acting agents (target TXA_2_S and TP) have shown great promise in reducing metastasis, slowing primary tumor growth and increasing host survival. Tumor growth was similar between mice treated with a higher dose of cisplatin (5mg/kg) and GR32191 [56]. Furthermore, mice injected with amelanotic melanoma cells (B16a) or lung cancer (3LL) cells had a significant reduction in metastatic lesions when 4 mg/kg of TXA_2_S antagonist, carboxyheptal imidazole (CI), was administered for 18 days, compared with the control [115]. In mouse breast cancer models (4T1), pan-TP antagonists (such as CPI211) prevent hematogenous metastasis in xenograft models of human breast, pancreatic and lung cancer [77]. TXA_2_S over expression and knockdown promoted and attenuated metastatic disease [69]. In addition, furegrelate increased the chemo-/radiosensitivity of glioma cells in mice to 1,3-bis(2-chloroethyl)-1-nitrosourea (BCNU) and γ-radiotherapy compared to chemo-/radiotherapy alone [96,116]. Similar increases in chemo-sensitivity were observed in bladder cancer xenografts when TXA_2_S inhibitors were used as adjuvant/neoadjuvant therapies *in vivo*.

Collectively, these data provide proof of principle for the robust anti-tumor effects of TXA_2_ antagonism. It is curious, therefore, that clinical trials have been so slow to embrace these agents as adjuvant/neoadjuvant therapies. Many clinical trials have looked to inhibit platelet function to prevent TXA_2_ signaling in cancer patients; however, (as outlined above) this thinking is flawed, as strategies to prevent platelet activation do not necessarily decrease TXA_2_ synthesis by the tumor. Indeed, TXA_2_ synthesis in tumors may be downstream of COX-2, not COX-1 as it is in platelets, so the strategies for cancer therapy need to be different. Whilst many trials have assessed the anti-tumor efficacy of COX-1 (aspirin) and COX-2 (Celecoxib) inhibitors on primary endpoints such as median survival, response rate, time to progression and distant metastasis (recently reviewed [6,7]) only one trial has undertaken a directed approach to specifically examine the effects of TXA_2_ antagonism on tumor progression. Clinically, TP antagonists are perhaps the best strategy for such a trial as TXA_2_, PGG_2_, and PGH_2_ (precursors that would accumulate with TXA_2_S inhibitors) also bind and activate TP [117]. The current trial (Clinicaltrials.gov #NCT03694249) will recruit patients (based at Vanderbilt-Ingram Cancer Center) to examine the effects of the orally-active TP antagonist Ifetroban (250 mg daily) in patients with malignant solid tumors at a high risk of recurrence after treatment and undergoing metastatic spreading. The trial is designed to examine the role of platelets in mediating distant metastasis; however, Ifetroban will also block TP activation on cancer cells. Thus, the trial will also assess ablation of tumor TP activation on metastatic potential and impact on event-free survival. With the trial not due to release results until 2025, we will wait for the outcome of this first in-human validation of the principles so potently demonstrated in preclinical and *in vitro* models.

## 7. Sleight of Hand: Looking beyond the Tumor Epithelium to Determine the Role of TXA_2_ in Cancer

**Figure 6:**
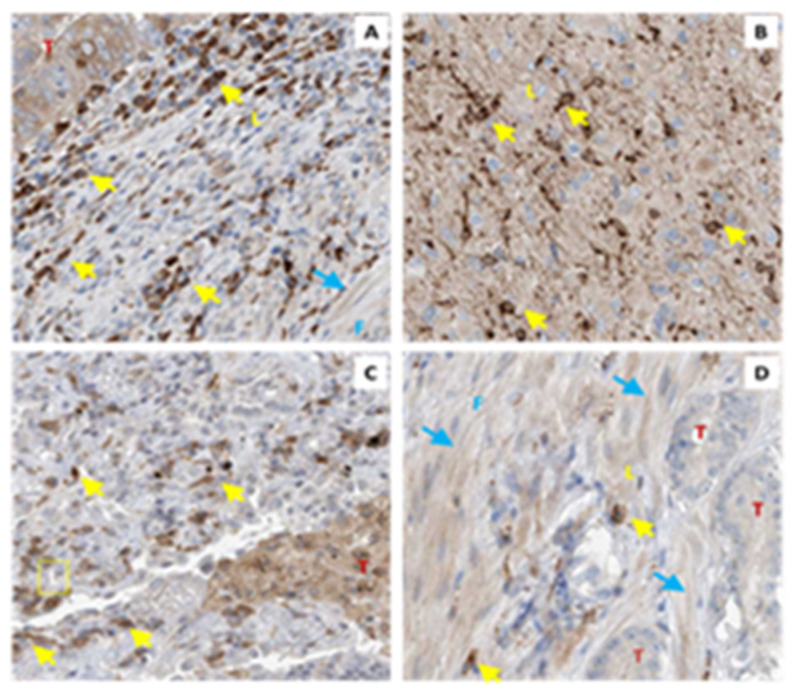
**TXA_2_S is Expressed in Tumor Stroma**. Biopsies from colorectal (**A**) brain (**B**), lung (**C**) and prostate (**D**) cancer were immunostained with antibodies against TXA_2_S. Tumor (T), leukocytes (L, ←) and fibroblasts (F, ←) all stain positively. Data are from the human protein atlas.

The concepts of tumorigenesis are constantly evolving from original ideas about oncogenes and loss of tumor suppressors to now include the interplay between cancer cells and the surrounding microenvironment. TXA_2_ signaling also shapes the tumor microenvironment by manipulating inflammation and immunity [92], angiogenesis [118], clotting and fibrosis/fibroblast infiltration. This is important, as cancer cells are not the only source of TXA_2_S in tumors with both tumor-associated leukocytes and stromal fibroblasts expressing significant TXA_2_S (Figure 6).

## 8. Immune Modulation by TXA_2_ Signaling in Cancer

While it has long been acknowledged that the immune system recognizes tumors as “foreign”, the concepts of immune invasion in cancer are more recent. Tumors have developed various strategies to avoid T cell-mediated killing of cancer cells. The role of TP activation in regulating T-cell subsets has been explored previously. TXA_2_ synthesis is associated with biased Th2 polarization of T-cells [118], providing a growth advantage to many tumor types [119]. Furthermore, TP activation induces thymocyte apoptosis *in vitro* and TP null mice are resistant to LPS-induced thymocyte apoptosis [120,121]. Physical interaction of T cells and dendritic cells (DCs) is essential for T cell proliferation and differentiation. DCs were found to produce TXA_2_, while naïve T-cells express TP [122]. The product of this heterotypic cell signaling is the increase in the random motion of naïve T-cells, which prevents DC-T cell adhesion, T-cell proliferation and lineage specification. In support of this, T-cell responses to foreign antigens are enhanced in the presence of TP antagonists and in TP null mice [122,123]. The high TXA_2_ release from tumor cells likely co-opts this regulatory mechanism to modulate anti-tumor immunity directly. Moreover, TP stimulation of Ca^2+^ transients by TP activation suppresses CD8+ T cells and cDC1 in Braf^V600E^ melanoma and 4T1 Breast cancer allografts [124]. This modulation of acquired immunity promotes immune evasion in the early stages of tumorigenesis and identifies TP signaling as an essential immunomodulator of the tumor microenvironment.

The microenvironment within solid tumors is often immunosuppressive; however, once tumor cells enter the bloodstream to facilitate metastasis, they are exposed to a normally operating immune system, able to attack and destroy tumor cells efficiently. Indeed, less than 0.1% of tumor cells injected into mice eventuate in metastases [125] and most cells die within 1–2 days [126]. Natural killer (NK) and cytotoxic (CD8+) T-cells play a central role in this type of tumor immune-surveillance and anti-tumor activity. In this case, TP stimulation has augmented cytotoxic activity in mixed lymphocyte populations and TP/TXA_2_S inhibitors diminish the response [127,128]. Moreover, CD8 expression was often increased, and proliferation increased, by TP stimulation of mixed lymphocyte populations.

Macrophages play critical roles in innate and adaptive immunity and are known for their remarkable phenotypic heterogeneity and functional diversity. Tumor-associated macrophages (TAMs) are recruited from circulating monocytes to tumors, where the tumor microenvironment influences them to either promote tumor resolution (M1/M(LPS)) or tumor growth, invasion, metastasis, and drug resistance (M2/M(IL-4))(recently reviewed [129]). TXA_2_ and 12-HHT are direct chemoattractants for monocytes [130] and TP stimulation of lung cancer promotes monocyte recruitment through the upregulation of MCP-1 and CCL2 expression, suggesting a role for TXA_2_ signaling in intra-tumoral recruitment of TAMs [92]. While TP directly stimulates monocyte/macrophage activation [131], the effect of TP stimulation on macrophage polarization has not been determined. However, platelet-derived TXA_2_ is thought to play a role in the M1 polarization of LPS-treated monocytes by activated platelets [132], which is inconsistent with differentiation along the M1 lineage being associated with loss of endogenous TXA_2_S and COX-1 expression [133]. These data suggest that TP signaling plays a prominent role in manipulating the tumor microenvironment, skewing it toward immune tolerance and tumor growth.

## 9. Role of TXA_2_ Signaling in Priming Sites of Metastatic Spread

**Figure 7:**
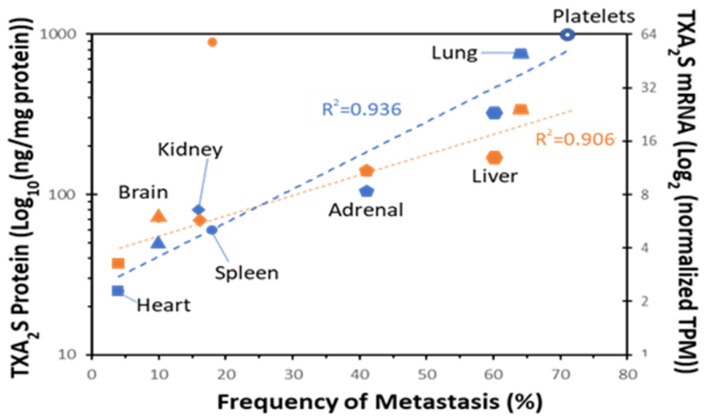
**TXA_2_S expression is elevated in common sites of metastasis.** TXA_2_S protein (blue) and RNA (Orange) expression was graphed against the frequency of metastasis from breast cancer (RNA expression from human protein atlas, metastasis data from [137]).

Factors influencing the site at which tumor cells lodge is especially pertinent to controlling metastasis. The association between TXA_2_S expression and metastatic burden has been recognized (Figure 7); however, the mechanism underlying this relationship is far from established. The first implication may be that high local TXA_2_ levels in a tissue act as an “exit ramp” for circulating tumor cells to extravasate from the vasculature. TXA_2_ is chemotactic for many cancer cells *in vitro* and locally elevated TXA_2_ production may be enough to promote adhesion to endothelial cells and movement out of the vasculature. TP stimulation on endothelial cells (especially TPβ) enhances expression of cell–cell adhesive molecules and promotes vascular permeability (opening of inter-endothelial junctions) that would facilitate this process [134,135]. In support of this concept, TXA_2_S inhibitors abrogate lung colonization by Lewis lung carcinoma or B16a cells after being intravenously injected in a C57Bl6 lung seeding model [116] and prevents endothelial activation, tumor cell adhesion to the endothelium, and recruitment of metastasis-promoting monocyte/macrophages [136]. Inhibition of TXA_2_ signaling diminishes the formation of pre-metastatic and intravascular metastatic niches through abrogation of platelet-tumor cell-endothelial cell interactions mediated through P-selectin-mediated aggregate formation [93]. However, if tumor cells are released and the “soil” they find themselves in is not hospitable, they will not develop into metastases. Thus, does TXA_2_ signaling condition the metastatic niche to create a more nurturing environment?

The effects of TP on cancer-associated fibroblasts have not been investigated; however, they are likely to be significant given the emerging role of these cells and TXA_2_ signaling in tumor progression. TP expression is upregulated in lung fibroblasts of mice and patients in response to damage and inflammation, as are isoprostanes [138]. TXA_2_ is also released by lung fibroblasts in response to bradykinin, phospholipase activation, IL-1β and thrombin *in vitro*, prompting the suggestion that lung fibroblast are likely the most prominent pulmonary producers of TXA_2_ *in vivo* [139,140]. Expression of TXA_2_S in lung cells is primarily driven by p300 and Nrf2 [141] which have been implicated in lung metastasis [142]. TP signaling in fibroblasts promotes proliferation, fibrosis and transformation to the myofibroblast phenotype, via potentiation of TGF-β signaling [138,143,144]. Increased fibrosis induces a switch in cancer cell growth via integrin/Src-mediated signaling, leading to cancer progression through the modulation of the pro-inflammatory niche [145]. Moreover, aged/senescent lung fibroblasts disproportionately increase TXA_2_ synthesis compared to “young” cells [146]. Senescent cancer-associated fibroblasts also induce cancer cell motility by suppressing PTEN [147], which is reversed by inhibiting COX-2. Thus, the pro-tumorigenic effects of the senescence-associated secretory phenotype may be mediated, in part, by TXA_2_. The TXA_2_ signaling may not only direct circulating tumor cells to accommodating sites for metastasis, but also prepare those sites before implantation and foster metastatic lesions’ development by creating protected environments designed to facilitate growth.

## 10. Role and Regulation of Endothelial Cell Migration and Angiogenesis by TXA_2_

The formation of new blood vessels (neovascularization) is essential to the development of all solid tumors, including tumor stage/grade, metastasis and recurrence rate. Multiple mechanisms (angiogenesis, vasculogenesis and intussusception) contribute to neovascularization in tumors (reviewed in [148]); however, the outcome of the neovascularization response is based on the balance of pro- and anti-angiogenic factors in the tissue. TP stimulation has both pro- and anti-angiogenic effects in multiple experimental systems. In endothelial cells, TP activation attenuates migration (58%) and angiogenesis (85%) [149] and ablates the response to both VEGF-A [150] and FGF-2 [151] *in vitro* and *in vivo*. Moreover, TXA_2_ signaling induces apoptosis in endothelial cells [152]. The downstream regulation is unique to each stimulus with attenuation of nitric oxide signaling, FGFR2 internalization and gap junction function responsible for the attenuation of VEGF, FGF-2 and spontaneous angiogenesis, respectively [149,150,151]. TP stimulation has also been reported to induce endothelial cell death associated with retinal vascular degeneration [153] and diabetes [154,155]. TP stimulation synergizes with platelet releasate [156] and neukinin B [157] to prevent angiogenesis. In addition, isoprostanes (8-iso-PGF2α, 8-iso-PGE2 and 8-iso-PGA2) inhibit coronary endothelial cell migration and differentiation from cardiac explants ex vivo by influencing actin remodeling without influencing apoptosis [158]. These data support the anti-angiogenic nature of TP stimulation in endothelial cells and that blocking TP activation would be beneficial in conditions where angiogenesis is lacking, such as myocardial infarction.

Conversely, TXA_2_S expression in tumor cells correlates with enhanced angiogenesis, shortened survival time and increased tumor growth rate [159]. However, the authors did not distinguish between direct effects on endothelial cells versus changes in tumor biology to produce new anti-regulatory molecules. In fact, TXA_2_ stimulation of lung cancer cells increases VEGF release, through TPα activation with subsequent downstream activation of ERK, PKA, EGFR and Src kinases, which would be integral to the neovascularization response [85,118]. However, evidence of increased neovascularization was only macroscopic and could have easily resulted from hemorrhage into the growing tumors. Direct pro-angiogenic effects of TP stimulation on the endothelium increase FGF-2 and VEGF expression up to 5-fold [115,160], stimulating the differentiation and migration of endothelial cells *in vitro*. TP signaling promotes spontaneous and growth-factor-induced neovascularization in the corneal and aortic ring explant assays, the rodent ovary and [115,159,161,162]. If these data hold, then the inhibition of TXA_2_ synthesis or TP signaling during diseases such as cancer would prevent vessel formation, slowing tumor growth and prolonging survival.

TPα and β have almost identical coupling to heterotrimeric G-proteins, resulting in misplaced complacency regarding their distinct roles in disease. Our data [135,149,151] and that of others [115,158,160] suggest isoform specific regulation as a basis for these dichotomous effects on angiogenesis. In fact, most of the anti-angiogenic effects of TXA_2_ have been demonstrated in human model systems that have the possibility of expressing TPβ. Our data also show that expressing TPβ in the endothelium from TP null mice inhibits migration and differentiation *in vitro*, and transgenic mice overexpressing TPβ in endothelial cells display reduced angiogenesis in Matrigel plug models [150,151]. The small animal models upon which the pro-angiogenic properties of TXA_2_ are largely based are flawed, as they do not express TPβ, a normal part of endothelial biology [16], due to the absence of the splice acceptor site in the *TBXA2R* gene [163]. Moreover, our data suggest that the anti-angiogenic properties of TPβ dominate in EC expressing both isoforms [150,151]. The primary deficit in the original report of the TPβ transgenic mouse was intrauterine growth restriction associated with reduced placental size [164]. One explanation for such a phenotype would be reduced neovascularization during development, resulting in attenuated placental and fetal growth. The isoform-specific regulation of angiogenesis by TPα and TPβ suggests that the divergent residues in the C-terminus are the basis of the selective mechanism. The tail of TPβ contains multiple sites that could regulate receptor signaling but few are well characterized. Certainly, the tail residues of TPβ have very different protein–protein interactions and signaling properties than those of TPα [39,47,48,49]. TPβ binding partners, such as angio-associated migratory cell protein (AAMP)(binds TPβ at residues 366–392), are implicated in RhoA activation and actin-based motility, which is important for angiogenesis [165]. Such isoform-specific binding partners, whilst currently untested for angiogenic potential, highlight the role of non-G-protein mediated mechanisms in regulating angiogenesis by TP isoforms and further strengthen the notion of divergent pathological roles for TPα and TPβ in diseases such as cancer, which are heavily reliant upon vascular remodeling.

## 11. Summary and Future Directions

Collectively, the above data represent a strong case for the importance of TXA_2_S and TP in cancer progression and metastasis. The evolution of TXA_2_ biology in cancer has progressed from an unknown COX-2 metabolite to a nuanced analysis of the role of individual isoforms, signaling unrelated to heterotrimeric G-proteins and beyond cancer cells to the immune and stromal response of the tumor. Thus, the roles of TP signaling encompasses all aspects of tumor biology that produce a tumor microenvironment which promotes carcinogenesis. These realizations are essential to progress the clinical utility of antagonists to this pathway as adjunct therapies for cancer prevention and treatment, as they have been adopted for the treatment of cardiovascular disease. The trial of TP antagonists as anti-cancer agents shows that the clinical utility of these compounds is finally being recognized. However, many questions remain, such as the role of individual isoforms in many cancers and the unrecognized contributions of TP signaling to many cancers with the highest expression of TP/TXA_2_S.

## Data Availability

Not applicable.
